# Structural basis for glucocorticoid receptor recognition of both unmodified and methylated binding sites, precursors of a modern recognition element

**DOI:** 10.1093/nar/gkab605

**Published:** 2021-07-21

**Authors:** Xu Liu, Emily R Weikum, Desiree Tilo, Charles Vinson, Eric A Ortlund

**Affiliations:** Department of Biochemistry, Emory University School of Medicine, Atlanta, GA 30322, USA; Department of Biochemistry, Emory University School of Medicine, Atlanta, GA 30322, USA; Laboratory of Metabolism, National Cancer Institute, National Institutes of Health, Bethesda, MD 20892, USA; Laboratory of Metabolism, National Cancer Institute, National Institutes of Health, Bethesda, MD 20892, USA; Department of Biochemistry, Emory University School of Medicine, Atlanta, GA 30322, USA

## Abstract

The most common form of DNA methylation involves the addition of a methyl group to a cytosine base in the context of a cytosine–phosphate–guanine (CpG) dinucleotide. Genomes from more primitive organisms are more abundant in CpG sites that, through the process of methylation, deamination and subsequent mutation to thymine–phosphate–guanine (TpG) sites, can produce new transcription factor binding sites. Here, we examined the evolutionary history of the over 36 000 glucocorticoid receptor (GR) consensus binding motifs in the human genome and identified a subset of them in regulatory regions that arose via a deamination and subsequent mutation event. GR can bind to both unmodified and methylated pre-GR binding sequences (GBSs) that contain a CpG site. Our structural analyses show that CpG methylation in a pre-GBS generates a favorable interaction with Arg447 mimicking that made with a TpG in a GBS. This methyl-specific recognition arose 420 million years ago and was conserved during the evolution of GR and likely helps fix the methylation on the relevant cytosines. Our study provides the first genetic, biochemical and structural evidence of high-affinity binding for the likely evolutionary precursor of extant TpG-containing GBS.

## INTRODUCTION

The most common type of DNA modification involves the covalent addition of a methyl group to the 5-carbon position of a cytosine base to produce 5-methyl cytosine (5mC), which almost always occurs in the context of a cytosine–phosphate–guanine (CpG) dinucleotide (1). Methylated CpGs are dispersed throughout the genome, but dense clusters of CpGs, termed CpG islands, are commonly found in gene promoters and are generally unmethylated (2). Context-specific DNA methylation in these regions is associated with a compact chromatin morphology and gene silencing (3). DNA methylation plays important roles in X-chromosome inactivation, genetic imprinting and suppression of transcription (1,4,5). Despite the importance of DNA methylation in regulating transcription, mammalian genomes contain relatively low numbers of CpGs that decrease roughly 4-fold compared to other dinucleotides (6,7). This is in contrast with invertebrate genomes, which have a higher CpG content (7,8).

The loss of CpGs in the evolution from invertebrates to vertebrates has been attributed to the relative ease of 5mC deamination to a thymine base, generating a T–G mismatched base pair (9,10). Though repair machinery is in place to correct this error, it is inefficient and often results in the T**–**G base pair mutating to a T–A base pair and thus a thymine–phosphate–guanine (TpG) site (11,12). The generation of a TpG from a 5mCpG site is thought to be the reason for the overall depletion of CpGs in mammalian genomes (13). In fact, there is an inverse relationship between CpG and TpG dinucleotides; genomes with low CpG enrichment tend to have a higher occurrence of TpG dinucleotides, and vice versa (13). Even before the vast availability of sequenced genomes, it was postulated that 5mC deamination and subsequent mutation to a T–A base pair could promote genetic diversity during evolution (6). A recent study hypothesized that mutation to a TpG could generate new transcription factor binding sites (TFBSs), as demonstrated for activator protein-1 (AP-1) response elements (TREs), **TpG**AG/CTCA, where the bolded TpG sites were derived from ancient CpG sites (8,14). This study found that genomes with plesiomorphic traits, such as those from coelacanth and *Xenopus*, contain a high abundance of CpG-containing TREs that are TpG sites in mammalian genomes (e.g. humans and mice) (8). The ability of a 5mCpG to mimic a TpG in ancestral TREs also plays a role in the dynamic generation of AP-1 binding sites in human genome and represents 2% of all AP-1 bound elements as detected by ChIP-seq (15). Structural characterization of Jun/Jun homodimers bound to an ancestral TRE with a 5mCpGAGTCA DNA sequence revealed that an alanine residue in the DNA-binding helix forms equivalent contacts with both 5mCpG and TpG (16). In addition to TREs, CpG to TpG substitutions were enriched during the evolution of tetrapods in nuclear receptor (NR) palindromic TFBS, such as the glucocorticoid receptor (GR) binding sequence (GBS), AGAACAnnnTGTTCT (8). Thus, we hypothesize that a subset of modern GBSs could have been generated upon deamination of methylated cytosine nucleotides.

The GR is a ligand-regulated transcription factor (TF) that controls the expression of thousands of genes (17). GR has a domain structure common to the NR superfamily: an unstructured N-terminal domain, a zinc finger (ZnF)-containing DNA-binding domain (DBD), a hinge region and a ligand-binding domain (18,19). To modulate transcription, GR binds directly to DNA at canonical GBSs composed of two pseudo-palindromic hexameric repeats separated by a 3-bp spacer (5′-AGAACAnnnTGTTCT-3′) (20). The canonical GBS contains no apparent CpG; however, the effect of DNA methylation on GR binding has been investigated (21,22). GR binds a canonical GBS as a dimer oriented in a head-to-head fashion (23–25). The mechanisms surrounding GR–GBS interactions are well studied and understood. Yet, how structurally GR could interact with a GBS with a 5mCpG in pseudo-palindromic hexameric repeats has not been explored.

Here, we set out to examine whether CpG→5mCpG→TpG transitions could have generated a subset of current GBSs by integrating bioinformatics, biochemistry and structural biology. We first identify GBSs that historically contained a CpG dinucleotide in the tetrapod lineage by examining the evolutionary history of GR consensus motifs in humans. Most of these motifs are associated with regulatory regions, indicative of functional GR motifs. Next, we biochemically characterize the extant human GR (hereafter GR, unless labeled otherwise) DBD bound to an unmethylated CpG-containing GBS (pre-GBS) and its methylated counterpart (5mC-GBS) and find that GR has preference for the 5mCpG GBS over the pre-GBS. Crystal structures of these complexes reveal a specific van der Waals interaction between Arg447 in GR DBD and the methyl moiety in 5mC-GBS sequences, which is absent in the GR DBD–pre-GBS complex structure. Importantly, this binding specificity governed by the methylation status is maintained throughout the evolution from ancestral steroid receptors (SRs) to extant GR. Collectively, our findings provide mechanistic and historical insights into how GR recognized methylated sites during the evolution of modern GBSs.

## MATERIALS AND METHODS

### Evolutionary analysis of the GR motif

To determine the evolutionary history of the GR motif, we identified all 36 899 occurrences of the motif GnACAnnnTGTnC in the University of California, Santa Cruz (UCSC) build hg19 of the human genome. Occurrences of the GR 13-mer in nine other genomes were then examined by extracting homologous regions from pairwise alignments of hg19 with nine other genomes obtained from the UCSC Genome Bioinformatics website (http://genome.ucsc.edu/) (26): mouse (mm9), dog (canFam3), elephant (loxAfr), opossum (monDom5), chicken (galGal3), lizard (anoCar2), frog (xenTro3), coelacanth (latCha1) and stickleback (gasAcu1). Occurrences of the GR motif in other genomes that did not contain any insertions or deletions were used for further analysis.

### Data sets

Genomic coordinates of all publicly available human DNase I hypersensitive sites (DHSs) from 125 tissue and cell lines and all available GR ChIP-seq peaks (six data sets from HepG2, ECC-1 and A549 cells) from the ENCODE Project Consortium (27) were obtained from the UCSC Genome Bioinformatics website (http://genome.ucsc.edu/) (26). In addition, we obtained genomic coordinates of GR ChIP-seq peaks in human U2OS (27), A13 (28) and breast cancer (29) cells and conserved non-coding elements (CNEs) (30). We used the BEDTools suite (31) to intersect each GR motif occurrence with each of these data sets in our analyses. A GR motif occurrence was classified as ‘regulatory’ if it overlapped any of the annotations (DHS, GR ChIP or CNE) in our analysis.

### Functional enrichment of extant GBSs

To examine the potential functions of deamination-derived GBSs in humans, we associated the genomic coordinate information of these GBSs with genes using the Genomic Regions Enrichment of Annotations Tool (GREAT) (32). We used the 200 bp surrounding these GBS motifs that are likely to be regulatory as input into the GREAT, using the full genome as the background set. GREAT robustly incorporates distal binding sites and uses a binomial test for bias elimination to associate genomic regions rather than genes, different from other procedures, for the enrichment analysis.

### Protein expression and purification

Ancestral DBDs were reconstructed by the maximum likelihood method as described previously (33,34). All SR DBD proteins were expressed and purified as described previously (34,35). Briefly, target genes were cloned with a 6X-histidine tag into the pMCSG7 vector and transformed in BL21 (DE3) pLysS *Escherichia coli*. These were grown in TB media at 37°C to an OD_600_ of 0.6 and then were induced with 0.3 mM IPTG and grown for additional 4 h at 32°C. Cells were lysed in 20 mM Tris–HCl (pH 7.4), 1 M NaCl, 25 mM imidazole and 5% glycerol via sonication. Protein was purified using affinity chromatography (His-Trap) followed by gel filtration chromatography. Protein was then concentrated to 3–4 mg/ml in 20 mM Tris–HCl (pH 7.4), 150 mM NaCl and 5% glycerol, flash frozen in liquid N_2_ and stored at −80°C.

### Nucleic acid binding assays

Sequences of DNA constructs used for fluorescence polarization assays were as follows: GBS: FAM-5′-TGAGAACAGAGTGTTCTTT-3′, 5′-AAAGAACACTCTGTTCTCA-3′; 5mC-GBS: FAM-5′-CCAGAACGGAG**C**GTTCTGA-3′, 5′-TCAGAACGCTC**C**GTTCTGG-3′ (where the bolded C is methylated); and pre-GBS: FAM-5′-CCAGAACGGAGCGTTCTGA-3′, 5′-TCAGAACGCTCCGTTCTG-3′. Synthesized FAM-labeled nucleic acid duplexes (Integrated DNA Technologies) were annealed by heating to 90°C followed by slow cooling to room temperature. Fluorescence polarization assays were performed by adding increasing concentrations of purified DBDs (1 nM to 50 μM) to 10 nM of the FAM-labeled DNA. All reactions were performed in 20 mM Tris–HCl (pH 7.4), 150 mM NaCl and 5% glycerol. Polarization was monitored on a BioTek Synergy 4 plate reader at an excitation/emission wavelength of 485/528 nm. Three technical replicates and three biological replicates were conducted and graphs are a compilation of all data collected. The program GraphPad Prism (v8) was used to analyze binding data and generate graphs. Binding data were analyzed by curve fitting to a one-site binding event, which generated dissociation values (*K*_d_) with its 95% confidence interval. Error bars represent standard deviation (SD) from three independent experiments conducted in triplicate.

### Structure determination of GR DBD–5mC-GBS and GR DBD–pre-GBS complexes

Crystals of the GR DBD–5mC-GBS complex were grown by hanging drop vapor diffusion in 50 mM sodium cacodylate (pH 6.5), 80 mM calcium chloride, 1% glycerol and 7% PEG 400 with a 2:1 protein:DNA molar ratio. Crystals were cryoprotected with 50 mM sodium cacodylate (pH 6.5), 80 mM calcium chloride, 30% glycerol and 30% PEG 400 and flash cooled in liquid N_2_. Crystals of the GR DBD–pre-GBS complex were grown by hanging drop vapor diffusion in 50 mM sodium cacodylate (pH 6.5), 80 mM calcium chloride, 1% glycerol and 8.5% PEG 400 with a 2:1 protein:DNA molar ratio. Crystals were cryoprotected with 50 mM sodium cacodylate (pH 6.5), 80 mM calcium chloride, 10% glycerol and 20% PEG 400 and flash cooled in liquid N_2_. Data were collected at 1.00 Å wavelength at the 22-ID beamline (Advanced Photon Source, Argonne, IL) and processed using the HKL-2000 software (36). The structures were phased using a previously solved structure of GR DBD–GBS complex (PDB 3FYL) in PHENIX (37). Structure refinement and validation was performed using PHENIX refine software and model building was performed in COOT (37,38). PDB Redo was used iteratively to optimize refinement parameters and geometry (39). PyMOL v1.8.2 was used to visualize structures and generate figures (Schrödinger, LLC).

## RESULTS

### Deamination events have generated a subset of functional human GBSs

**Figure 1.**
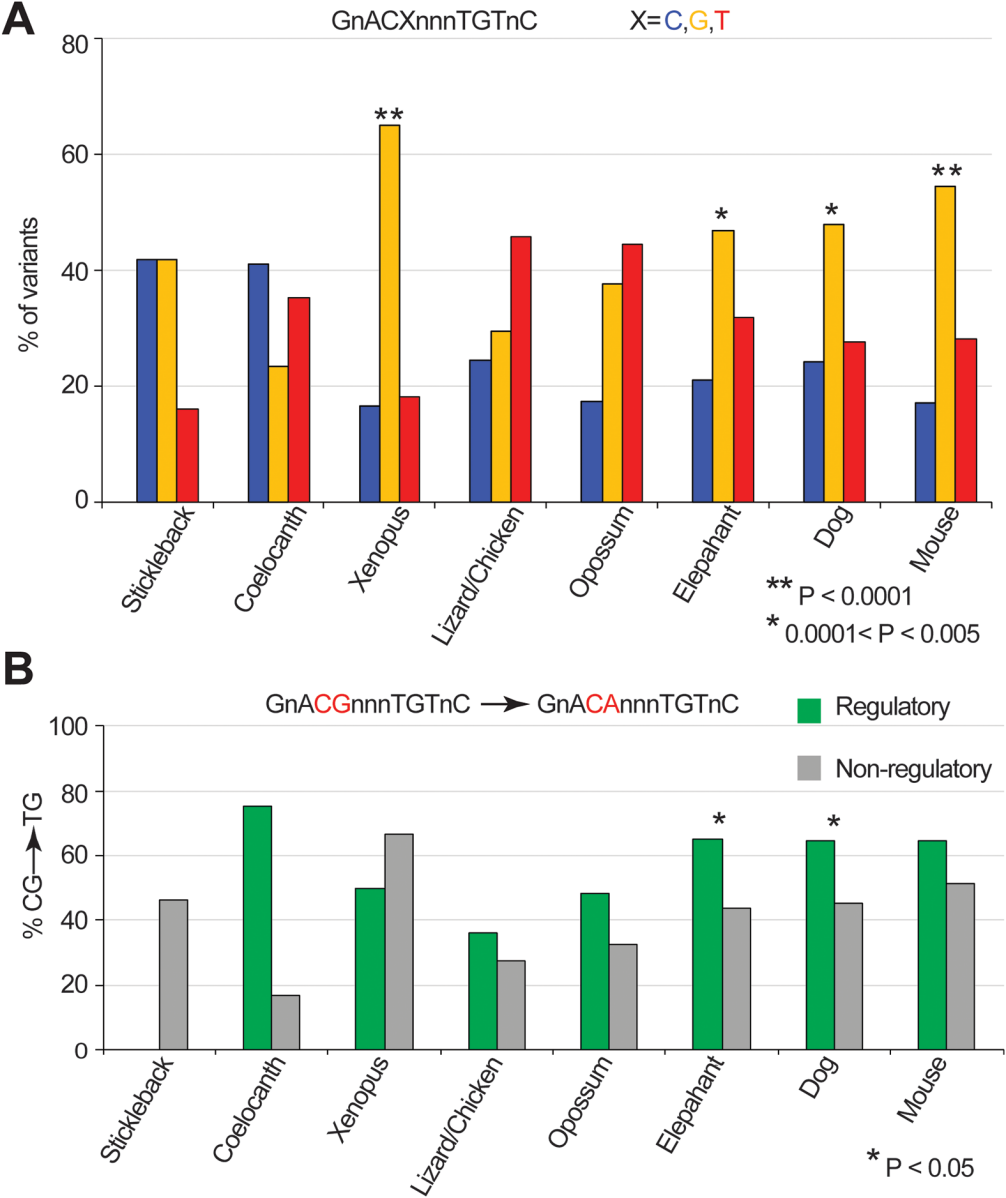
Deamination of cytosines generates functional GR motifs. (**A**) Proportion of single-nucleotide variants of the human GR motifs in other genomes from stickleback to mouse being C, G or T at position 5 (GnACXnnnTGTnC). Asterisks denote genomes with significant CG→CA transition (**P < 0.0001; 0.0001 < *P < 0.005; chi-square test). (**B**) Percentage of nucleotide variations in different genomes with cytosine at position 5 of the human GR motif. Occurrences are classified into two sets: those that overlap with CNEs, DHS or GR ChIP-seq (‘Regulatory’, green) and those that do not (‘Non-regulatory’, gray). Asterisks denote genomes with significant CG→CA frequency differences between regulatory and non-regulatory regions (*P* < 0.05; Fisher’s one-sided test).

We asked whether GBSs are derived from sequences that previously contained a CG dinucleotide by examining the evolutionary history of the 36 899 GR motifs in the human genome. To this end, we mapped all occurrences of the GR motifs (GnACAnnnTGTnC) to the genomes of nine different species (see the ‘Materials and Methods’ section), encompassing all major tetrapod lineages from stickleback to mouse, and found >59 000 homologous GR motifs (Supplementary Table S1). To examine the subset of GR motifs that may have arisen due to deamination of CG dinucleotides, we constrained our analysis to homologous sequences in other species containing a single base variant at position 5 or 9 (GnAC**A**nnn**T**GTnC) (Supplementary Tables S2 and S3). A variant at position 5 (GnAC**X**nnnTGTnC) on the forward strand would be a variant at position 9 (GnACAnnn**X**GTnC) on the reverse strand; therefore, we compute occurrences of variants of position 5 of the GR motif on both strands. Additionally, since both cytosines in a CG dinucleotide are typically methylated, deamination of a single CG to TG on one strand will give a complementary CA dinucleotide on the other strand. We find that GnAC**G**nnnTGTnC is the most frequent variant in the *Xenopus*, elephant, dog and mouse genomes (Figure 1A). In particular, GnAC**G**nnnTGTnC variants comprise over 50% of the variants at position 5 in *Xenopus* and mouse genomes. Overall, we find 1017 total pre-GBSs that contain variations at these positions, among which there are 883 unique pre-GBSs after removing those appearing more than once in different species.

**Figure 2.**
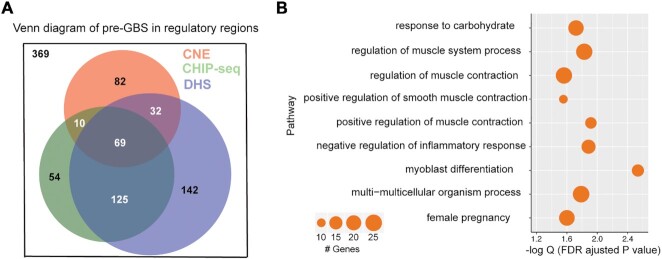
Majority of deamination-derived GR motifs are functional. (**A**) Venn diagram of a subset of 883 GBSs that overlap with CNE (red), GR ChIP-seq (green) or DHS (purple), with a total number of 514. (**B**). Gene ontology pathway enrichment with significant features (false discovery rate adjusted binomial *P*-values). Circle size is proportional to the number of significant genomic regions in each pathway, ranging from 10 to 25.

We next examined whether deamination-derived pre-GBS resulted in potentially functional GR sites (i.e. those that overlap a regulatory region defined by a ChIP-seq, CNE or DHS site; see the ‘Materials and Methods’ section). We find increased occurrences of CG→CA pre-GBSs in regulatory regions compared to non-regulatory regions in elephant and dog genomes (*P* < 0.05) (Supplementary Table S4). Most other species (except for *Xenopus*) show similar trend (Figure 1B). In particular, 71% of regulatory pre-GBSs identified in the dog genome previously contained a C at position 5 of the GR motif (GnAC**G**nnnTGTnC), whereas only 44% contain a C at this position in non-regulatory regions (Supplementary Table S2). This could be partially due to generally high GC content and thus higher chance of deamination in regulatory regions than those in non-regulatory regions. However, we expect natural selection to play a predominate role in preserving newly created and functional GR sites. Overall, of the 883 human GR motifs that may have arisen from deamination events, 514 (58%) overlap with a regulatory region. This overlap is statistically significant (hypergeometric *P*-value: 5.4e−53), and provides evidence that deamination of pre-GBSs resulted in functional binding sites (Figure 2A). Gene ontology enrichment analysis of deamination-derived GR sites indicates that they are enriched for biological processes related to muscle function, inflammation and metabolism (Figure 2B and Supplementary Figure S1).

### GR binds to a 5mC-GBS and an unmethylated pre-GBS

**Figure 3.**
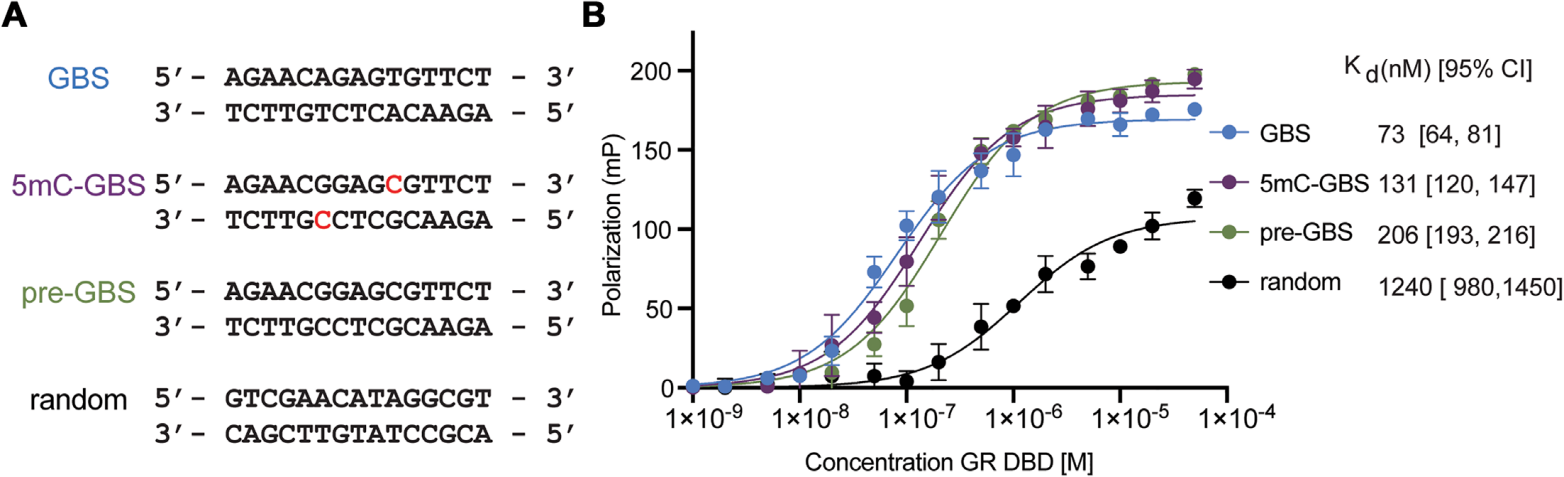
Specificity of GR binding to pre-GBS, methylated pre-GBS and extant GBS. (**A**) DNA sequences used in the binding assays, with methylated cytosines highlighted in red. (**B**) GR binds to three GBSs with different affinities as monitored by a fluorescence polarization assay. Error bars in (B) indicate SD from three replicates and from three independent experiments. Binding affinities are presented as the mean [95% confidence interval] from these experiments.

The position of the methylated C in a 5mC-GBS sequence is not predicted to alter the GR–DNA hydrogen bonding pattern in structures observed to date (25). To empirically determine whether GR is capable of recognizing these pre- and methylated GBSs, from which the modern GBS is derived, we monitored the ability of recombinant GR DBD to bind a FAM-labeled canonical GBS, a 5mC-GBS and a pre-GBS via fluorescence polarization (Figure 3A). Similar to previous reports, GR DBD bound a canonical GBS with an apparent *K*_d_ of 73 [64, 81] nM (95% confidence interval) (34,35). Binding to the 5mC-GBS and pre-GBS showed *K*_d_ values of 131 [120, 147] and 206 [193, 216] nM, respectively (Figure 3B). All these are tighter than the GR binding to a random DNA sequence (non-specific binding) (Figure 3B).

### Structural analysis of GR in complex with 5mC-GBS and pre-GBS

**Figure 4.**
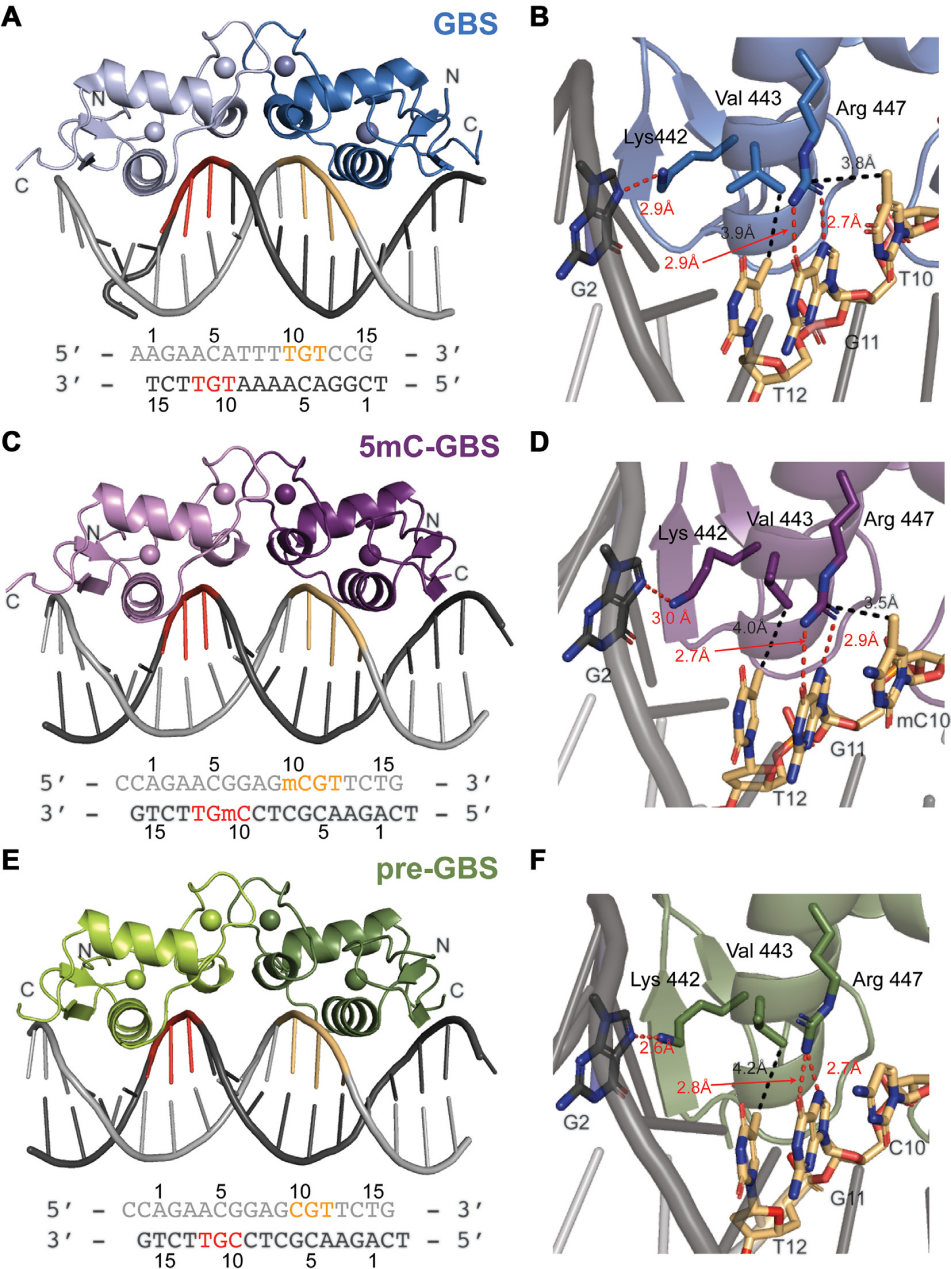
Structures of GR DBD bound to three GBSs. Overall structures of GR DBD in complexes with a canonical GBS (**A**), a methylated pre-GBS (**C**) and a pre-GBS (**E**). Two monomers of GR DBD are shown in light and dark colors, respectively. Forward and reverse strands of the DNA are shown in light and dark gray, with the residue numbers labeled and strand-specific TGT/mCGT/CGT highlighted in orange and red, respectively. GR base-specific interactions are shown in panels (**B**), (**D**) and (**F**). Hydrogen bonds and van der Waals interactions are colored in red and black dashed lines, respectively.

To determine how GR recognizes the 5mC-GBS and pre-GBS, we solved crystal structures of the GR DBD–5mC-GBS and GR DBD–pre-GBS complexes at a resolution of 2.0 and 2.5 Å, respectively (Table 1). Both complexes crystallized in the *C* 1 2 1 space group and each structure contains a dimer of DBD protein molecules in the asymmetric unit (Figure 4). Both structures show GR–DNA interactions characteristic of a canonical GR–GBS complex (Figure 4A and B) (23). GR binds in a head-to-head fashion creating interactions between dimeric GR DBDs (Figure 4C and E). The GR DBD utilizes its ‘DNA reading helix’—in particular, side chains of Arg447, Lys442 and Val443—to make base-specific contacts within the major groove of each GBS half site (Figure 4B, D and F). Arg447 makes hydrogen bonds to the 7-position amine and 6-position carbonyl on a guanine (G11). It also establishes a van der Waals contact with the 5-position methyl group of cytosine (mC10) in the 5mC-GBS structure (Figure 4D). This van der Waals interaction is missing in the pre-GBS structure, as the analogous cytosine (C10) remains unmethylated (Figure 4F). Val443 makes similar van der Waals contacts to methyl groups in thymine (T12) in both pre-GBS and 5mC-GBS, as observed in the GBS interaction. In all structures, Lys442 hydrogen bonds to the 7-position amine on a guanine (G2) on the opposite strand (Figure 4B, D and F and Supplementary Figure S2).

**Table 1.** Summary of crystal data collection and refinement statistics

	GR DBD–pre-GBS	GR DBD–5mC-GBS
**Data collection**		
Space group	C121	C121
Unit cell dimension		
*a*, *b*, *c* (Å)	*a* = 130.4, *b* = 39.0, *c* = 96.8	*a* = 130.4, *b* = 39.1, *c* = 97.4
*α*, *β*, *γ* ( °)	90, 118.7, 90	90, 118.6, 90
Resolution (Å)^a^	2.480 (2.569–2.480)	2.001 (2.072–2.001)
*R*_pim_	0.079 (0.297)	0.076 (0.584)
CC $\frac{1}{2}$	(0.705)	(0.692)
*I*/*σ*	14.05 (3.99)	16.5 (1.40)
Completeness	98.51 (97.92)	98.69 (90.22)
Redundancy	3.1 (2.8)	6.7 (4.0)
**Refinement**		
No. of reflections	15 305 (1504)	29 195 (2639)
*R*_work_/*R*_free_	18.95/20.37	18.11/21.22
No. of atoms		
Protein	1127	1117
DNA	730	692
Water	13	30
*B*-factors		
Protein	66.98	64.22
DNA	86.68	88.50
Water	65.20	55.76
**RMS deviations**		
Bond lengths (Å)	0.005	0.007
Bond angles (°)	0.67	0.88
**Ramachandran plot (%**)		
Most favored	94.4	96.5
Outliers	0	0

^a^Values in the parentheses are for the highest-resolution shell.

### Comparison of the GR DBD–pre-GBS and GR DBD–5mC-GBS to other GR DBD–GBS complexes

**Figure 5.**
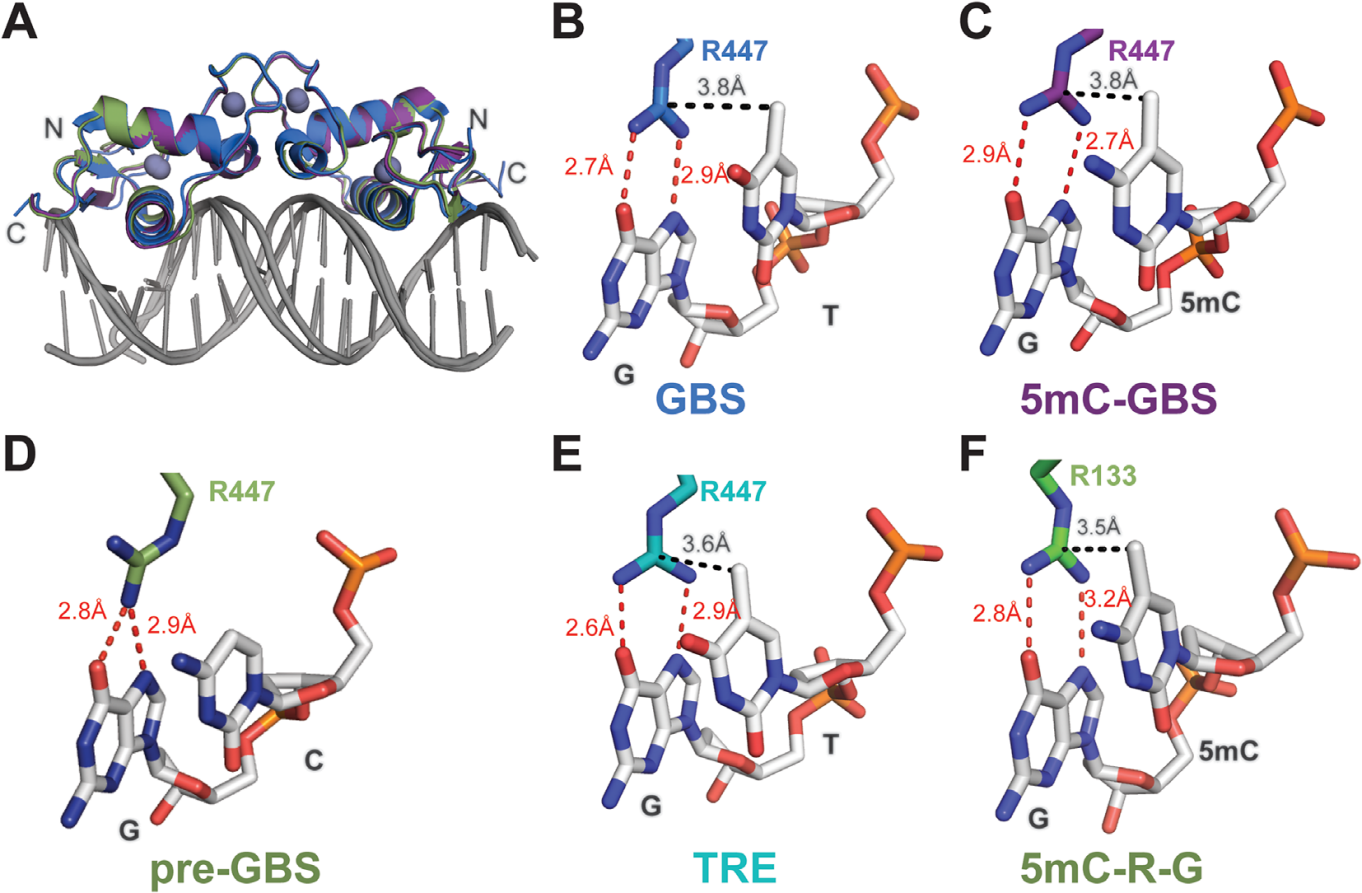
Structural comparison of GR binding to canonical GBS, pre-GBS, modified pre-GBS and TRE sites. (**A**) Structural overlay of GR bound to GBS (blue), 5mC-GBS (purple) and pre-GBS (green). Arg447 is involved in the base-specific hydrogen bonds (red dashed line) and van der Waals contacts (black dashed line) with TpG in GBS (**B**), methylated CpG in 5mC-GBS (**C**), unmodified pre-GBS (**D**), TpG in TRE (**E**) and the conserved 5mC–Arg–G triad as shown in MeCP2/DNA binding (PDB code: 3C2I) (**F**).

The overall structures of the GR DBD–pre-GBS and GR DBD–5mC-GBS complexes look almost identical to the canonical GR DBD–GBS structure (rmsd < 1 Å; Figure 5A) (23). Of note, the GBS used for crystallization has a different spacer sequence (–TTT–), compared to the 5mC-GBS and pre-GBS structures. The –TTT– spacer was previously shown to slightly narrow the minor groove (25), which is shown on the overlay with 5mC-GBS and pre-GBS structures that have (–GAG–) spacer sequence (Figure 5). DNAshape analysis confirmed a narrower minor groove width in the ‘–TTT–’ spacer region (Supplementary Figure S3) (40). We then compared the sequence-specific contacts beyond the spacer sequence between the known GR DBD–GBS complex and our new structures. Most base-specific interactions are maintained in all the three structures. However, unique interactions related to the methyl moiety, i.e. the van der Waals interactions, appear to be the molecular determinants for the enhanced binding affinity (Figure 3B). We found that in the 5mC-GBS structure, Arg447 makes side-on hydrophobic contacts with the methyl group of the 5mC, mimicking interactions with a thymine base seen in extant GBSs (Figure 5B and C). This hydrophobic interaction is lost in the GR DBD–pre-GBS complex that contains an unmodified cytosine base at this position. Without this interaction, Arg447 in monomer B moves outward but still makes hydrogen bonds with the guanine in CpG dinucleotide by one amide group (Supplementary Figure S2C and Figure 5D), suggesting the methyl-derived side-on contact helps stabilize the Arg447. This gain of a hydrophobic contact could explain the increase in affinity from the unmodified pre-GBS to methylated pre-GBS (Figure 3B). The detailed nucleotide/amino acid interactions analyzed by DNAproDB further corroborate this hypothesis (Supplementary Figure S4) (41). Each GR DBD monomer contacts the methylated pre-GBS with one more van der Waals contact than with an unmethylated pre-GBS, which results in larger buried solvent accessible surface area (BASA) between GR DBD and nucleotides in each half-palindromic site (140 Å^2^ versus 109 Å^2^ and 125 Å^2^ versus 115 Å^2^) (Supplementary Figure S4B and C). Deamination of a methylated CpG produces a TpG site and an even larger BASA (130 Å^2^ versus 125 Å^2^ and 152 Å^2^ versus 140 Å^2^) (Supplementary Figure S4A). The increase in BASA on the second half-palindromic site is mostly due to more van der Waals contacts formed between V443 and TG**T**_12_C of the top strand and **G**_3_ACA of the bottom strand (Supplementary Figure S4A), which likely contribute to the further improvement in affinity from a methylated pre-GBS to a GBS. Interestingly, replacing 5mCpG by TpG in an extant GBS also provides better DNA geometry as calculated by 3DNA (42). TpG has fewer deviations from standard B-DNA geometry (i.e. stretch, stagger, buckle and opening) than a 5mCpG; this also holds true for base-pair step and helical parameters (Supplementary Table S5).

The hydrophobic contact between GR Arg447 and 5mCpG methyl appears to play a critical role not only in canonical GR DBD–GBS recognition, but also in its binding to other DNA sites such as the TRE in the upstream of inflammatory genes interleukin (IL)-6 and IL-11 (43). Recently, our lab showed that GR is able to drive transrespression from these elements via direct DNA binding (44,45). The GR DBD binds to TREs in a tail-to-tail orientation on opposite sides of DNA, akin to inverted repeat GBS (IR-GBS) recognition (35). Arg447 from GR’s recognition helix makes similar interactions with thymine and guanine residues in the TRE site (Figure 5E). This highlights the importance of Arg447 as the key residue in GR–DNA recognition. Moreover, this suggests that the side-on Arg–DNA base methyl interaction permits recognition of methyl cytosine or thymine, in the context of CpG or TpG.

### Ancestrally reconstructed NR DBDs bind to pre-GBSs

**Figure 6.**
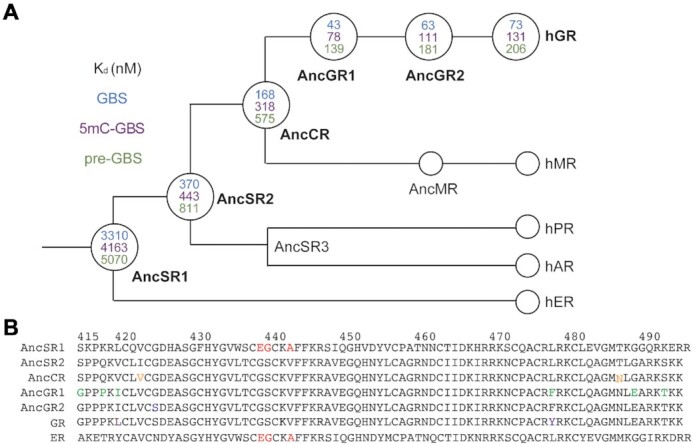
Binding specificity on GBS, methylated pre-GBS and pre-GBS during the evolution of different extant human SRs. (**A**) Simplified cladogram showing the evolutionary relationship between extant human SRs with binding affinities indicated in the nodes (open circles) of the tree. (**B**) Sequence alignment of AncSR1 and its daughter genes with substitutions highlighted by different colors.

The GR, androgen receptor, progesterone receptor, mineralocorticoid receptor (MR) and estrogen receptor (ER) are closely related SRs; the first four receptors all recognize steroidal ligands containing a keto group on carbon 3 and thus are known as 3-keto SRs (46). All 3-keto SRs recognize the canonical GBS to drive transactivation; however, ER binds a different response element sequence and cannot transactivate from a GBS (34,47). To determine whether the ancestral SRs could bind and possibly favor the conservation of CpG- to TpG-containing GBSs, we tested the ability of ancestral SR DBDs to bind to the canonical GBSs, 5mC-GBS and pre-GBSs. These ancestral SRs are representative proteins that existed prior to and during the split of jawless and cartilaginous fish from teleosts and tetrapods (48). AncSR1 is the ancient ER-like SR and does not bind to any sequence tested with *K*_d_ values in the μM range (Supplementary Figure S5). AncSR2 is the precursor to modern 3-keto SRs and binds a canonical GBS (34). It also weakly binds to unmethylated and methylated pre-GBS, with *K*_d_ values above 800 and 400 nM, respectively. AncCR, the precursor to the corticosteroid receptors GR and MR, binds to pre-GBS and 5mC-GBS, with a slightly tighter affinity than the AncSR2 (575 [530, 619] nM versus 811 [755, 872] nM and 318 [283, 345] nM versus 443 [412, 475] nM, respectively). There is a dramatic increase in the binding affinity to all three sequences from AncCR to AncGR1, the last common ancestor of jawed vertebrate GR. AncGR1 binds to an unmodified and methylated pre-GBS with affinities of 139 [128, 155] and 78 [69, 89] nM, respectively, which is a 5-fold increase from AncCR. AncGR2 is also capable of binding to the pre-GBS and 5mC-GBS variants, yet with a weaker affinity compared to AncGR1. However, the AncGR2 binding to these sequences is still marginally tighter than the extant human GR (Figure 6A), which mirrors the trend observed for the canonical and IR-GBS (34). Overall, the binding affinity improves during 3-keto SR evolution while the specificity (modern GBS > CpG GBS > preGBS) remains unaffected, suggesting that AncSRs and AncGRs may have exerted evolutionary pressure to enrich TpG GBSs over CpG GBSs in functional genomic sites.

## DISCUSSION

Cytosine methylation at the C_5_ position is the most important covalent modification in DNA, occurring predominantly at CpG sites. This modification within the promoter region of genes plays a key role in genomic imprinting and X-chromosome inactivation and its dysfunction is highly associated with various human diseases, including cancers (49,50). CpG methylation also plays crucial roles in evolution. Once methylated, 5mC can be deaminated to thymidine, which occurs 10–50 times faster than the equivalent process on an unmodified cytosine (9,10). Inefficient DNA repair in vertebrates leads to the formation of a TpG dinucleotide after DNA replication and overall TpG excess with CpG deficit (11,12). Indeed, CpG dinucleotides are present ∼5-fold less frequently than what is expected based on the overall GC content in human and mouse genomes, whereas 2-fold less in zebrafish genome (7,51). A recent large-scale study using systematic evolution of ligands by exponential enrichment (SELEX) to investigate how 5mC impacts TF DNA binding specificity found that methylation on the CpG site can increase its binding to GR, a phenomenon observed in >30% of examined TFs. However, methylation of the CpG site does not increase preference for GR over TpG (21). We hypothesized a subset of extant GBSs may have evolved from a putative pre-GBS with a CpG site. We showed stepwise increased affinities for GR–pre-GBS interaction after methylation and deamination, in line with SELEX results (Figure 3) (21).

The affinity between GR and pre-GBS (206 nM) suggests the pre-GBS may be a functional glucocorticoid response element (GRE) as it is a variation of TpG-containing GBS. Indeed, these CpG-containing sequences have been identified as the secondary GBSs (21), found in the functional GREs characterized in 3134 cells (22), and other genomes, such as GREs for sgk gene in dog genome and ddit4 gene in human and mouse genomes (52). Another large-scale study on how sequence modulates GR transcriptional output identified a CpG-containing GRE variant (GnACAnnn**C**GTnC) is at least as active as the conventional GRE (GnACAnnn**T**GTnC) (53). Additionally, a recent study showed that the total effect of a C-to-T transition (C–T) on ATF4–DNA interaction can be decomposed into a C to 5mC (C–5mC) and a 5mC to T transition (5mC–T) (54). Our GR binding data also indicate that a 5mC-containing site can act as an intermediate of a C–T transition. Together, these results suggest that a subset of extant GBSs are molecular fossils of methylated and deaminated ancient pre-GBSs, provide a more ideal DNA geometry for GR binding and fix what was once a reversible DNA modification through a C–T transition.

Our work highlights the importance of a methyl-specific GR–DNA interaction, which has been overlooked in previous structural GR studies. A recent study focused on GBS methylation on non-CpG sites (AGAA**C**AnnnTGTT**C**T). However, minimal variation in structures after methylation and no direct contribution from the added methyl group to GR binding was observed as these modified nucleotides were not contacting GR (55). Since the identification of methyl-binding proteins (MBPs) (56), there has been a growing list of TFs that can recognize methylated DNA, including the C2H2 ZnF proteins, basic helix–loop–helix, basic leucine–zipper, homeodomain families and tumor suppressor protein p53 (54,57–61). A published analysis of 60 protein–DNA structures containing 5mCs that interact with amino acid side chains identified a methyl–Arg–G triad as a common mechanism employed by TF for readout of methylation, as demonstrated in MBP, C2H2 ZnF and p53 (54,58,61–64) (Figure 5F). The methyl group from the cytosine in this triad makes van der Waals interactions with the guanidino group of Arg, which stacks in between cytosine and its adjacent guanine and in turn hydrogen bonds with the guanine O6 and N7 atoms in a bifurcated manner (65). We show that GR utilizes a similar methyl–Arg–G triad, suggesting that evolution has leveraged a readily available side-on interaction with the Arg residue engaged in recognizing the G base edge in a CpG dinucleotide. The methyl group in this triad in many cases can come from a thiamine as exemplified in our structures of GR DBD–GBS, GR DBD–TRE and other TFs, such as Kaiso, Zfp57 and C/EBPβ (65,66). This suggests GR is a ‘methyl group only’ reader whereby the binding specificity at a certain position in the DNA sequence is determined by only a methyl group (64).

AncSR2 is the common ancestor of all 3-keto SRs and gained the ability to recognize pre-GBS and GBSs through the evolution of three key residues in the DNA recognition helix, E439G, G440S and A442V (AncSR1 numbering) (Figure 6A). AncSR2–pre-GBS binding is weak, suggesting that it would not have served as a strong driver to select for TpG enrichment in the modern GBS. This required transition through AncCR (generating I423V and T487N substitutions) to AncGR1 harboring six additional substitutions (i.e. S415G, Q418P, V420I, L478F, G489E and S492T), which shows 5-fold tighter binding to 5mC-GBS and pre-GBS compared to AncSR2 (Figure 6B). Our previous study showed that the V420I mutation significantly increased the binding of AncSR2 DBD to GBS (41 nM versus 125 nM), even though it does not directly interact with GBS (34). We believe this mutation plays a similar role in the recognition of ancestral and methylated GBSs during evolution. Interestingly, I420L is found in the hGR, which might be associated with its weaker binding to GBS compared to AncGR1. Further investigation should be focused on the role of these allosteric residues in fine-tuning GBS binding during the evolution of the receptor–DNA relationship.

AncGR2 responds exclusively to cortisol and distinguishes cortisol- from aldosterone-mediated signaling pathways. It evolved roughly 420 million years ago and was first found in the ancestor of tetrapods and ray-finned fish (48). An increased genomic frequency of TpG-containing TFBSs, created due to the loss of efficient mismatch repair, was first observed in coelacanth that evolved roughly 400 million years ago (8). Therefore, AncGR2 DBD–DNA binding preferences (i.e. TpG over CpG) evolved prior to the genomic enrichment of TpG-containing binding sites. Indeed, the key ‘methyl reader’ residue R447 is maintained throughout the evolution from the AncSR2 to AncGR2 to modern GR. The alternative situation would be that GR DBD ancestors prefer CpG over TpG and over evolutionary time they gradually switch to the TpG preference. This would require more complicated evolutionary trajectory for both the DNA sequences and proteins, particularly substitutions on those residues physically contacting with the DNA. Our result is in line with concept of ‘molecular exploitation’ where an existing protein, previously constrained for a different role (such as CpG recognition here), fortuitously has affinity for a closely related ‘off-target’ molecule (such as 5mCpG here) and can be recruited into a new functional complex (i.e. recognizing TpG-containing TFBS) (67). Our results showed how a DNA methylation event can generate a transient (perhaps lower affinity) DNA binding site that can become permanent through a deamination event and suggest a potential role for AncGR2 in fixing the reversible modification (5mCpG). This parallels hormone specificity observed in AncSR2 and AncCR, whereby hormone binding preferences emerged earlier than the hormone itself, co-opting a steroid ligand into a new signaling pathway (48,68).

Together, our findings provide genomic, biochemical and structural evidence that a subset of extant GR DNA binding sites may have evolved from a CpG-containing pre-GBS site via methylation, subsequent deamination and mutation. Further studies utilizing similar strategies are essential to illustrate the molecular mechanisms of epigenetic contribution to the evolution of other TFs and their binding sites, particularly those that do not harbor CpG sites in their core consensus sequences.

## DATA AVAILABILITY

The atomic coordinates and structure factors have been deposited in the Protein Data Bank with the accession numbers 6X6D and 6X6E for GR DBD–pre-GBS and GR DBD–5mC-GBS complexes, respectively.

## Supplementary Material

gkab605_Supplemental_Files
