# Supplementary material for: Structural basis for glucocorticoid receptor recognition of both unmodified and methylated binding sites, precursors of a modern recognition element
Source: Nucleic Acids Res. 2021 Jul 21;49(15):8923–33. doi: 10.1093/nar/gkab605 (PMC8421226; doi:10.1093/nar/gkab605)
Supplement: gkab605_Supplemental_Files [file gkab605_supplemental_files.zip › GR-MethylC-XL-resubmission-NAR-supple-v2.pdf]

Supplementary Data

Manuscript title: Structural basis for glucocorticoid receptor recognition of both unmodified and methylated binding sites, precursors of a modern recognition element

Xu Liu[1#], Emily R. Weikum[1#], Desiree Tilo[2], Charles Vinson[2] and Eric A. Ortlund[1*]

[1] Department of Biochemistry, Emory University School of Medicine, Atlanta GA 30322 USA

[2] Laboratory of Metabolism, National Cancer Institute, National Institutes of Health, Bethesda, MD 20892, USA

[#] These authors contributed equally to this work as first authors

* To whom correspondence should be addressed.: Tel: 404-727-5014; Fax: 404-727-2738; Email: eortlun@emory.edu

5 figures and 5 tables

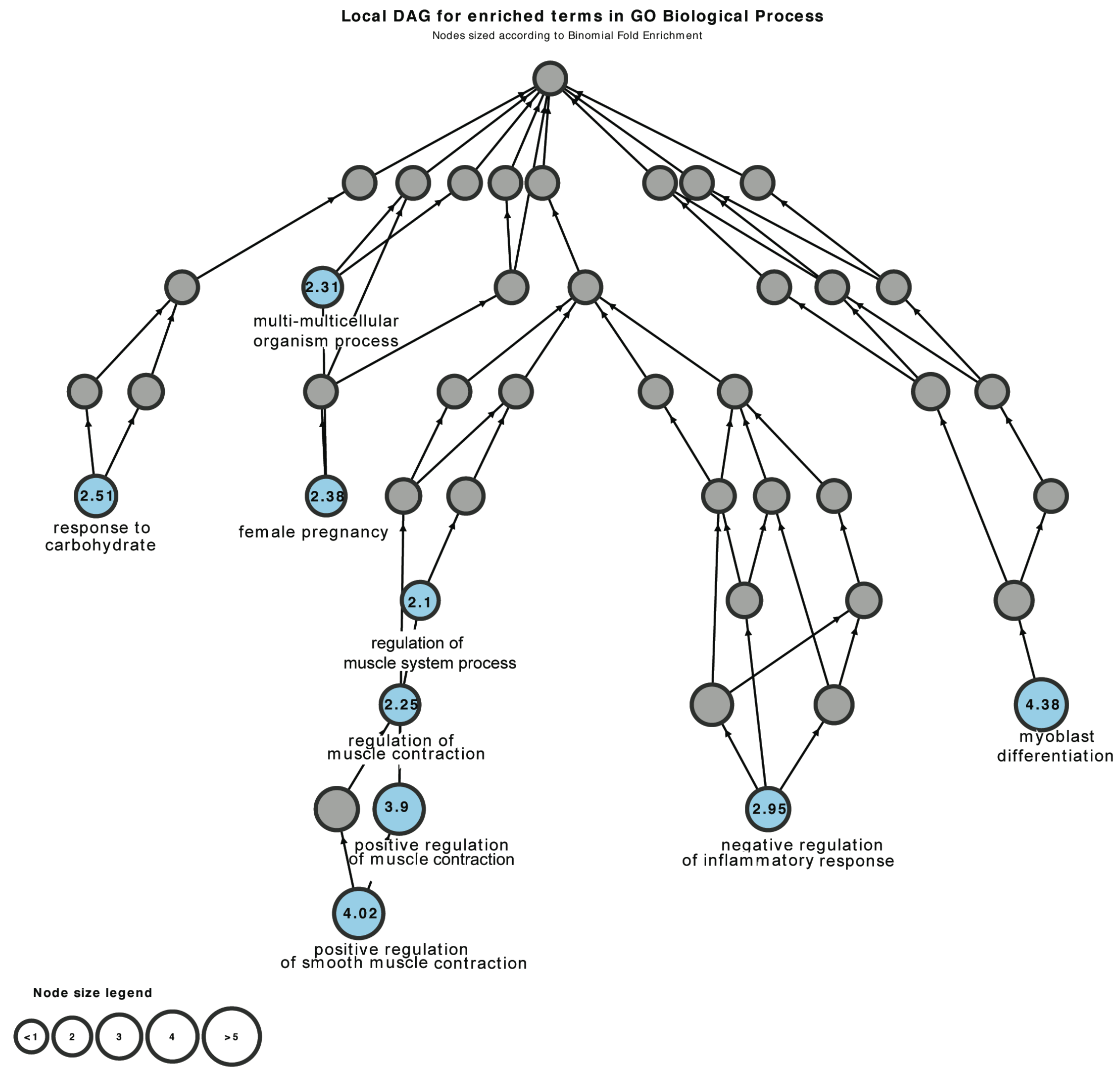

Supplementary Figure 1. A directed acyclic graph (DAG) showing the gene ontology hierarchy based on the enriched terms from a single ontology-specific table obtained from GREAT. Enriched terms are shown in cyan blue. Nodes have been sized according to Binomial Fold Enrichment.

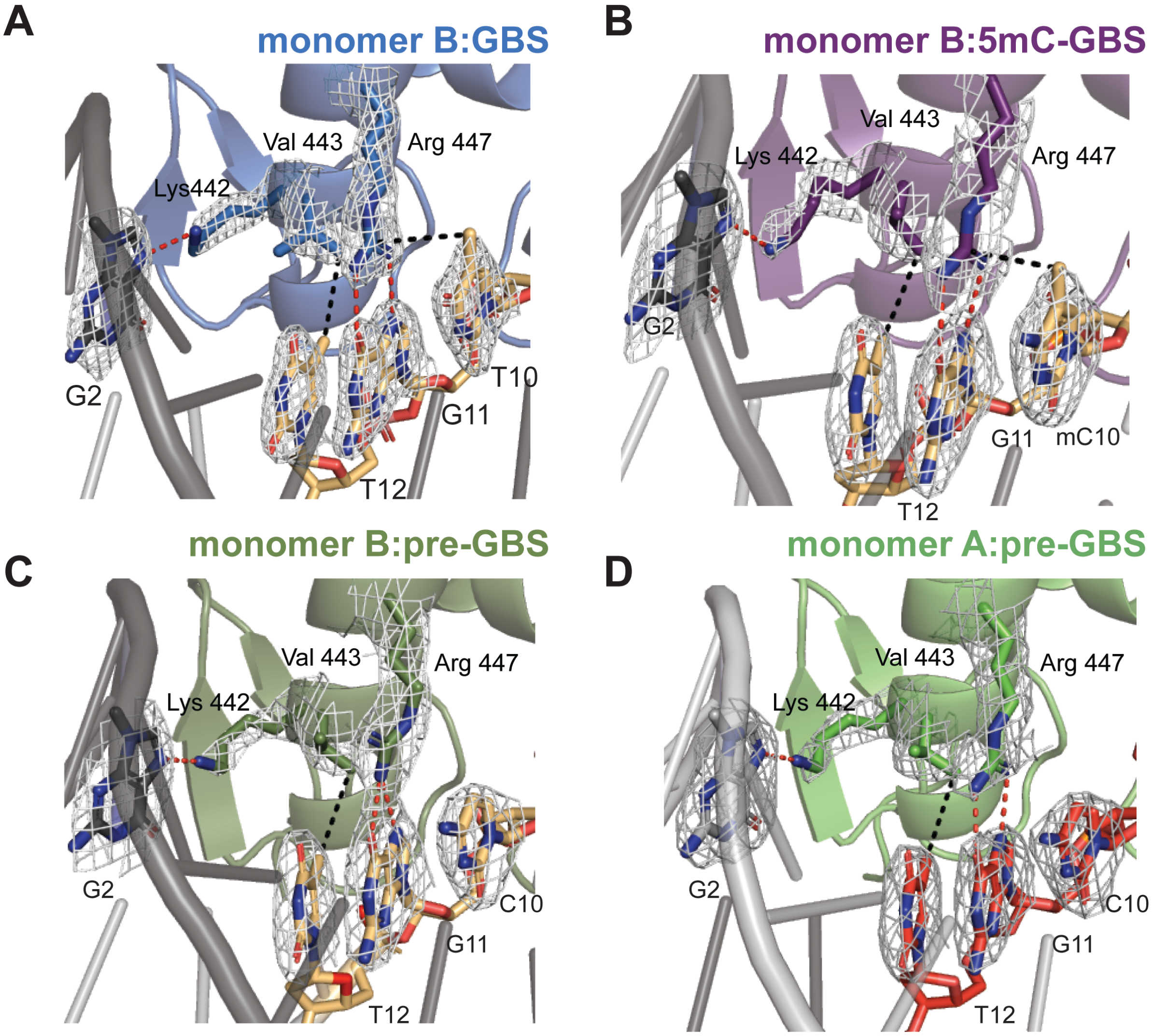

Supplementary Figure 2. The 2Fo-Fc omit electron density map (contoured to 2.0 σ) surrounding side chains of amino acids and nucleobases participating hydrogen bonding and Van der Waals interactions shown in Figure 4.

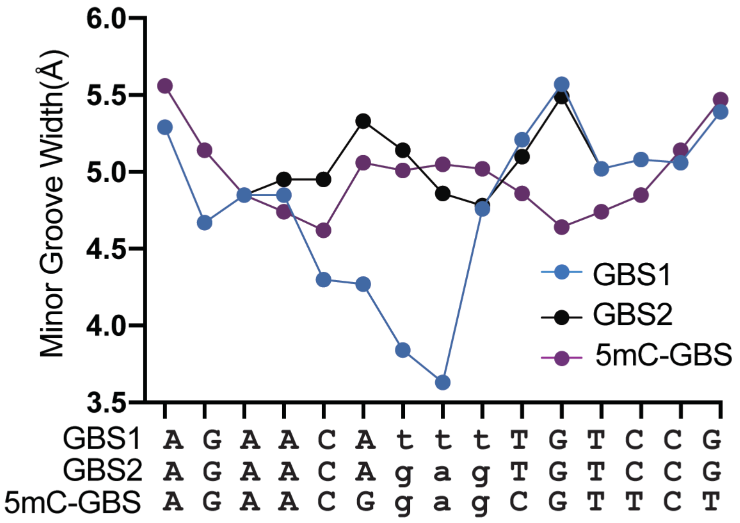

Supplementary Figure 3. Comparison of minor groove width of three GBSs with different spacer sequences analysed by DNAshape.

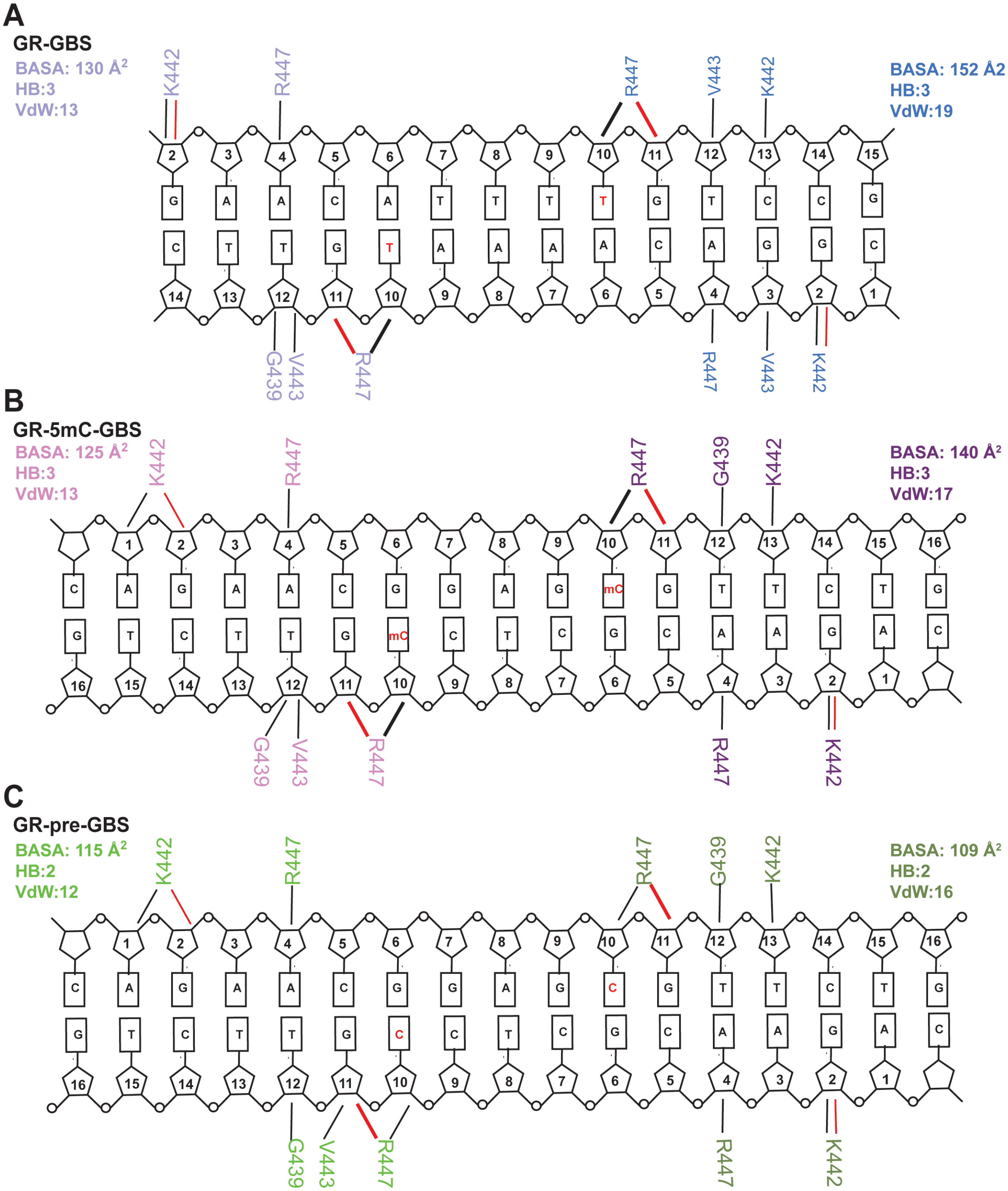

Supplementary Figure 4. Comparison of detailed GR/DNA interactions between GR-GBS (**A**), GR-methylated pre-GBS (**B**) and GR-unmodified pre-GBS (**C**). Interactions with chain A and B of GR DBD are colored in green and blue, respectively. Numbers of hydrogen bonds (HB), Van der Waals (VdW) and buried solvent accessible surface area (BASA) for GR/DNA interactions at each half-palindromic site are shown. HB and VdW interactions are colored in red and black lines, respectively, with two HBs or VdW interactions shown in bold.

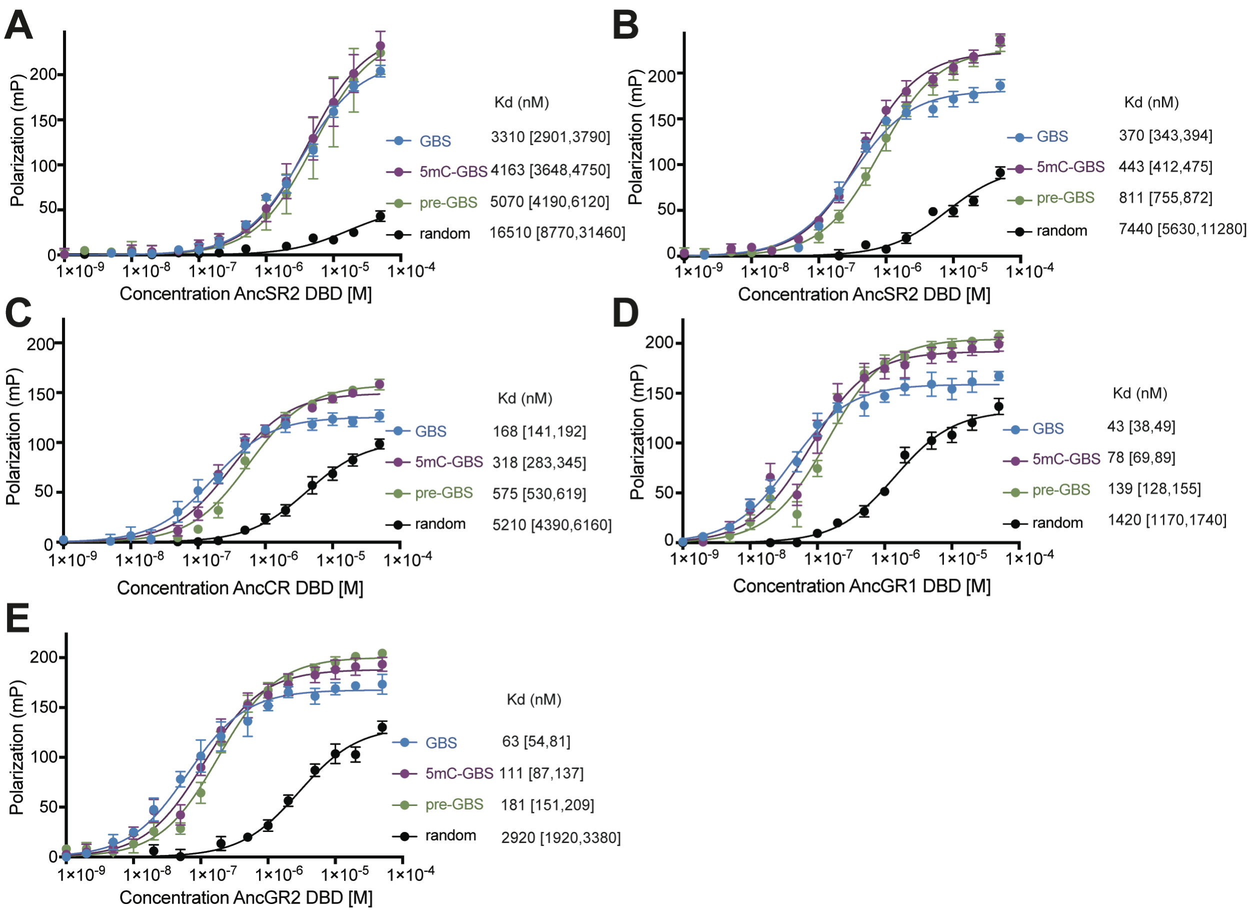

Supplementary Figure 5. Specificity of AncSR1 (**A**), AnsSR2 (**B**), AncCR (**C**), AncGR1 (**D**) and AncGR2 (**E**) DBD binding to pre-GBS, 5mC-GBS and extant GBS. Error bars indicate S.D. from three replicates and from three independent experiments. Binding affinities are presented as the mean [95% confidence interval] from these experiments.

Supplementary Table S1. GR motifs in human mapped to 9 tetrapod species

Group	Species	Genome Assembly	all		2-mismatches		1-mismatch	
			# of homologous GR sequences	% of Human GR motifs	# of homologous GR sequences	% of Human GR motifs	# of homologous GR sequences	% of Human GR motifs
01.Osteichthyes	Stickleback	gasAcu1	904	2.4%	483	1.3%	240	0.7%
02.Sarcopterygii	Coelacanth	latCha1	1,015	2.8%	575	1.6%	358	1.0%
03.Tetrapods	Xenopus	xenTro3	1,252	3.4%	635	1.7%	368	1.0%
04.Amniotes	Chicken	anoCar2	1,499	4.1%	800	2.2%	500	1.4%
04.Amniotes	Lizard	galGal3	1,487	4.0%	808	2.2%	510	1.4%
05.Theria	Opossum	monDom5	5,039	13.7%	2,180	5.9%	1,455	3.9%
06.Eutheria	Elephant	loxAfr3	17,561	47.6%	10,731	29.1%	6,716	18.2%
07.Boreoeutheria	Dog	canFam3	18,746	50.8%	11,337	30.7%	7,096	19.2%
08.Euarchontoglires	Mouse	mm9	11,507	31.2%	5,599	15.2%	3,478	9.4%

Supplementary Table S2. Base percentages of variants at each position for all GR motifs containing a single mismatch in dog (canFam3).

Position	1	2	3	4	5	6	7	8	9	10	11	12	13
**Consensus**	**G**	**N**	**A**	**C**	**A**	**N**	**N**	**N**	**T**	**G**	**T**	**N**	**C**
pre-GBS overlapping with CNE/DHS/ChIPseq (+) (1,269)													
**A**	71%	39%	--	20%	--	26%	32%	26%	28%	71%	8%	23%	19%
**C**	11%	22%	12%	--	17%	18%	18%	28%	**58%**	13%	85%	18%	-
**G**	--	19%	80%	13%	**71%**	27%	19%	17%	13%	--	8%	20%	9%
**T**	18%	21%	7%	67%	13%	29%	32%	28%	--	16%	--	39%	72%
pre-GBS overlapping with CNE/DHS/ChIPSeq (-) (5,827)													
**A**	71%	33%	--	19%	--	23%	27%	27%	28%	63%	13%	20%	17%
**C**	12%	27%	19%	--	26%	20%	23%	30%	**46%**	20%	68%	22%	--
**G**	--	22%	64%	18%	**44%**	29%	22%	20%	25%	--	19%	25%	13%
**T**	18%	18%	17%	63%	30%	27%	29%	24%	--	17%	--	33%	70%

Note: The base percentage of each component at that position is reported. "--" denotes the consensus motif. Percentages in **bold** highlight variations that result in CG dinucleotides.

Supplementary Table S3. Base percentages of variants at each position for all GR motifs containing a single mismatch in all nine species (see attached excel file).

Supplementary Table S4. Comparison of occurrences of CG->CA variations relative to all variations at position 5 (GnACXnnnTGTnC) shows an increased trend in regulatory than non-regulatory regions.

	**Non-regulatory**		**Regulatory**		**p-value**
Species	Number of sites with CG->CA variations	Number of sites with all variations	Number of sites with CG->CA variations	Number of sites with variations	(Fisher's one-sided test)
**Stickleback**	13	28	0	3	1.000
**Coelocanth**	5	30	3	4	0.113
**Xenopus**	36	54	3	6	0.768
**Lizard/Chicken**	20	73	9	25	0.356
**Opossum**	31	96	25	52	0.138
**Elephant**	253	580	69	106	0.013*
**Dog**	260	576	65	101	0.028*
**Mouse**	138	270	57	88	0.139

Supplementary Table S5. Base-pair parameters of 5mCpG-, TpG-containing GBS and corresponding B-DNA

Position*	Local base-pair parameters					
	Shear	Stretch	Stagger	Buckle	Propeller	Opening
5mCpG-5	-0.07	**-0.10****	**0.36**	**11.03**	-12.39	**1.91**
TpG-5	**0.05**	-0.19	0.29	9.69	-10.12	-1.90
B-DNA-5	-0.14	-0.18	0.10	0.26	-15.15	-1.30
5mCpG-9	0.11	**-0.01**	**0.61**	-16.50	-11.03	**2.57**
TpG-9	**-0.23**	-0.04	0.46	-15.24	-11.27	0.54
B-DNA-9	0.14	-0.18	0.10	-0.26	-15.15	-1.31
	Local base-pair step parameters					
Position	Step	Shift	Slide	Rise	Tilt	Roll
5mCpG-5	**-0.46**	-0.36	3.51	-0.60	**4.82**	36.61
TpG-5	-0.22	-0.35	3.50	0.06	3.96	36.04
B-DNA-5	0.01	0.47	3.36	-0.03	1.71	35.96
5mCpG-9	**-0.41**	0.10	2.89	**1.91**	4.37	29.66
TpG-9	-0.29	0.09	2.97	1.36	4.37	31.52
B-DNA-9	0.00	0.41	3.34	0.00	1.70	33.92
	Local base-pair helical parameters					
Position	X-disp	Y-disp	h-Rise	Incl.	Tip	h-Twist
5mCpG-5	-1.26	**0.65**	3.44	**7.63**	0.94	36.92
TpG-5	-1.16	0.36	3.45	6.38	-0.10	36.25
B-DNA-5	0.52	-0.02	3.37	2.76	0.04	36.00
5mCpG-9	-0.59	1.13	2.84	8.46	**-3.70**	30.04
TpG-9	-0.55	0.75	2.94	7.99	-2.48	31.84
B-DNA-9	0.43	-0.00	3.35	2.91	-0.00	33.96

* the position of the base pair is shown as below:

5mCpG: GAACG$_5$ (nnn) mC$_9$GTTCT;   TpG: GAACA$_5$ (nnn) T$_9$GTTCT

**5mCpG sites that result in larger deviations from B-DNA than TpG site are bolded.
